# Supplementary material for: Splice-Junction-Based Mapping of Alternative Isoforms in the Human Proteome
Source: Cell Rep. Author manuscript; Available in PMC 2020 Jan 15. (PMC6961840; doi:10.1016/j.celrep.2019.11.026)
Supplement: 2 [file NIHMS1546469-supplement-2.zip › DF1/PXD010154/Thyroid/Thyroid_20_PPFIBP1_KAAALEQMDGIIAGSK.pdf]

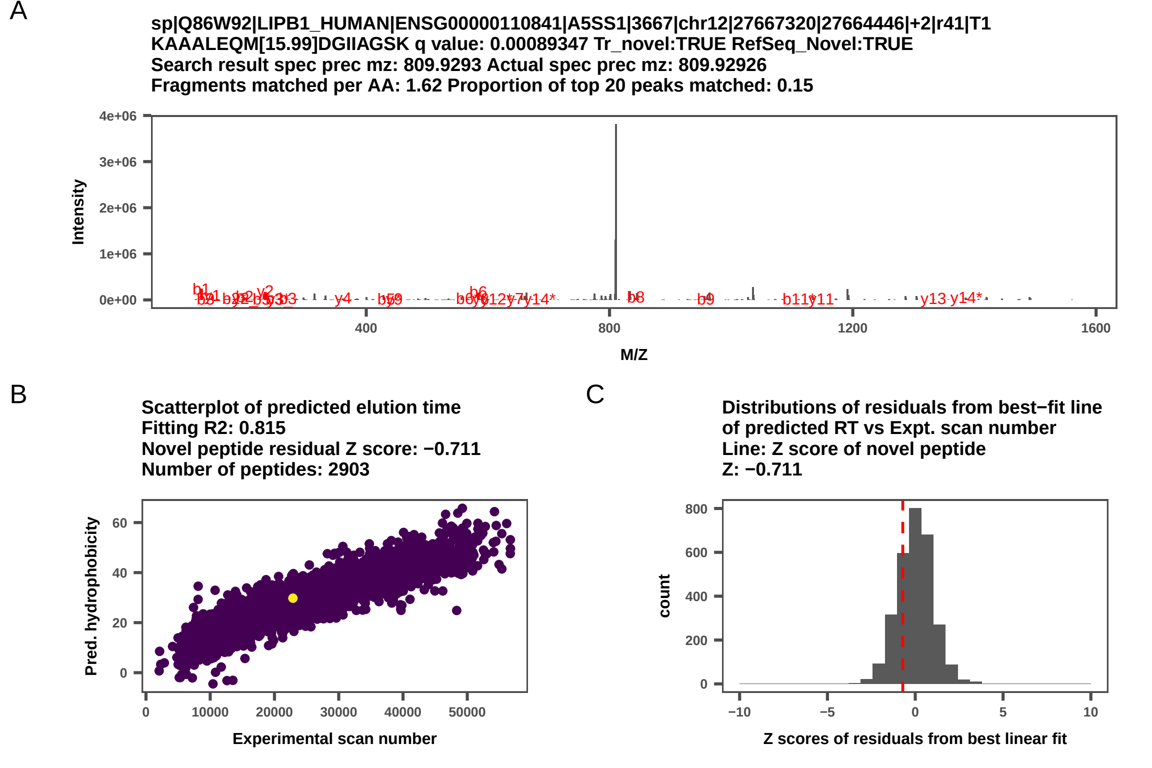
A
sp|Q86W92|LIPB1_HUMAN|ENSG00000110841|A5SS1|3667|chr12|27667320|27664446|+2|r41|T1
KAAALEQM[15.99]DGIIAGSK q value: 0.00089347 Tr_novel:TRUE RefSeq_Novel:TRUE
Search result spec prec mz: 809.9293 Actual spec prec mz: 809.92926
Fragments matched per AA: 1.62 Proportion of top 20 peaks matched: 0.15
Intensity
4e+06
3e+06
2e+06
1e+06
0e+00
y4
b8
b9
b11
y11
y13
y14*
400
800
1200
1600
M/Z
B
Scatterplot of predicted elution time
Fitting R2: 0.815
Novel peptide residual Z score: −0.711
Number of peptides: 2903
Pred. hydrophobicity
60
40
20
0
10000
20000
30000
40000
50000
Experimental scan number
C
Distributions of residuals from best−fit line
of predicted RT vs Expt. scan number
Line: Z score of novel peptide
Z: −0.711
count
800
600
400
200
0
−10
−5
0
5
10
Z scores of residuals from best linear fit
